# Mallory Denk Body Formation in Alcoholic Hepatitis: The Pivotal Role of Interleukin-8 Signaling

**DOI:** 10.4172/2327-5073.1000235

**Published:** 2016-02-25

**Authors:** Hui Liu, Samuel W French

## Description

Mallory-Denk Bodies (MDBs) are prevalent in various liver diseases including alcoholic hepatitis (AH) and are formed in mice livers by feeding diethyl 1,4-dehydro-2,4,6-trimethyl-3,5-pyridine-dicarboxylate (DDC). The chemokine CXCL8, also known as interleukin-8 (IL-8) and its receptors are involved in oncogenesis and in tumor progression, invasion, and metastasis. We reported previously the marked upregulation of IL-8 signaling in AH and DDC fed mice with MDBs present by RNA sequencing (RNA-Seq) analyses. Central molecules including IL-8 and the chemokine (C-X-C motif) receptor 2 (CXCR2) of this pathway were significantly upregulated in the livers of DDC re-fed mice and human liver biopsies from AH livers. MDB containing balloon hepatocytes in AH livers have increased intensity of staining of the cytoplasm for both IL-8 and CXCR2. Taken conjointly, these data indicates a crucial role of IL-8 signaling during MDB formation, and IL-8 and CXCR2 may be targeted as biomarkers for personalized treatment of AH.

The mortality and morbidity of alcoholic hepatitis (AH) is high. In one study the survival for 1 and 5 years was 82% and 40% respectively compared to non-alcoholic controls (99% and 96%). Treatment for severe AH patients can improve survival for 1 month but not 3 or 6 months [1]. The cost of hospitalization for AH is very high. In one study (Medicare and Medicaide paid 52% of AH) the cost averaged $37,769/hospitalization [2]. Reactive oxygen and nitrogen species (ROS and RNS, respectively) are products of normal cellular metabolism and oxidative stress has been proposed to be crucially involved in AH [3,4]. Oxidative stress caused by ROS is implicated in carcinogenesis and is used strategically to treat human cancer. The accumulation of indices of oxidative stress (e.g., lipid peroxides) are predominantly a hepatocellular event during alcohol administration. The proposed major sources of pro-oxidants in hepatocytes are ethanol-inducible CYP2E1, mitochondria and NADPH oxidase (NOX). In chronic liver disease, activation of NOX by hepatotoxic agents (e.g., bile salts) activate death pathways such as CD95-induced signaling to induce cell apoptosis [5], resulting in inflammation and fibrogenesis.

Mallory-Denk bodies (MDBs) are composed of intracellular aggregations of misfolded proteins in ballooned hepatocytes [6,7], and the pathogenesis of MDBs is associated with the development of ballooning of hepatocytes. MDBs are found in 70% to 75% of patients with alcoholic liver disease (ALD) [8,9]. A major player that determines MDB formation is oxidative stress. MDB formation is due to the failure of the 26S proteasome protein quality control system which leads to aggresomes composed of cytokeratins (CKs) and undigested proteins such as heat shock proteins (HSPs), ubiquitin (Ub), proteasome subunits, tubulin, and the ubiquitin-binding protein p62 [10,11]. In the DDC fed mouse model where liver cells proliferate, MDBs form and later, after DDC withdrawal (DDC primed hepatocytes), hepatocellular carcinoma (HCC) develops [12,13].

The first high-throughput RNA sequencing analysis (RNA-Seq) from Illumina was performed to explore the mechanisms (e.g., signaling pathways) that mediate the initiation and progression of liver MDB formation [14]. Ingenuity pathway analysis (IPA) demonstrated that a total number of 19 pathways were defined with at least three genes expressing each pathway [14], where IL-8 signaling was the 9th markedly changed pathway in AH livers with MDBs.

Deregulation of IL-8 signaling is shown to play pivotal roles in tumorigenesis and progression. IL-8 was originally identified as a potent neutrophil activator and chemotactic factor secreted by activated monocytes and macrophages. The biological effects of IL-8 are mediated via two classes A, rhodopsin-like guanine-protein-coupled receptors (GPCRs): CXCR1 (IL-8RA) and CXCR2 (IL-8RB). Many other cell types including fibroblasts, lymphocytes, neutrophils, endothelial cells, and a variety of normal and malignant epithelial cells have since been shown to secrete IL-8 [15]. IL-8 and CXCR1 direct neutrophil migration across the epithelial barrier into the lumen. Indeed, mIL-8Rh knockout mice showed impaired transepithelial neutrophil migration, with tissue accumulation of neutrophils, and these mice developed renal scarring [16]. Interestingly, CXCR2 knockout mice had fewer polymorphonuclear neutrophils (PMNs) in the colon but the other variables were unaffected except for an increase in colony-forming units (CFUs) in the colon [17]. However, whether MDBs would disappear or reduce in density or in number when IL-8 or IL-8r was knocked out or their mRNAs were inhibited needs to be further explored. It requires specific gene knockout mice to determine whether these key components are really involved in the MDB formation.

In order to explore the mechanisms (e.g., signaling pathways) that mediate the initiation and progression of AH, we used the normal liver controls to compare gene expression with AH biopsies which had formed MDBs. In all the cases liver forming MDBs were presented. Normal control livers were used for comparison. Also the AH livers without MDBs were used for comparison (n=3). At the same time, the livers of mice in which MDB formation was induced by feeding DDC were used to compare with the AH livers where MDBs had formed [13,18,19]. The genomic profile was compared using RNA sequencing (RNA-Seq) in a group of archived AH liver biopsies who had formed MDBs and archived normal liver controls with normal hepatic function.

Using Ingenuity Pathway Analysis (IPA), we found the marked change of IL-8 signaling in AH livers where MDBs had formed. It was also observed that IL-8 and CXCR2 mRNAs were also significantly up regulated in AH livers and DDC re-fed mice livers, which MDB containing balloon hepatocytes in AH livers had increased intensity of staining of the cytoplasm for both CXCR2 and IL-8 [18]. These observations suggested that the altered regulation of IL-8 signaling might result in MDB formation in AH. Recent advances reveal IL-8 signaling as a potential key to targeting breast cancer stem cells [19], suggesting that IL-8 and its receptors may be attractive targets for cancer therapy. So far we have shown using FAT10 KO mice that MDB formation require the increased expression of FAT10 meaning that the pathway of MDB formation is due to a deficiency of the 26S proteasome activity which causes the accumulation of undigested protein in the MDB forming liver cells [20].

IL-8 is upregulated in a wide variety of solid cancers, such as prostate, gastric, bladder, ovarian, lung, and melanoma, and is reported to contribute to multiple hallmarks of cancer, such as increased proliferation, angiogenesis, invasion, and metastases. Transcriptional activation of IL-8 is controlled primarily by NFκB, and IL-8 is one of the dominant transcriptional targets of inflammatory signaling mediated by NFκB, which is commonly activated in cancer cells [21]. The prominent up regulation of expression of FAT10 in the livers of AH patients was observed [11]. FAT10 expression is induced by interferon (IFN)-γ and tumor necrosis factor α (TNFα) in tumor cells [22,23], which indicate IL-8-mediated receptors may sustain NFκB activity by a feedback system in chronic inflammatory-associated microenvironments such as in AH [18].

Owing to the pleiotropic effects of IL-8, caution must be exercised when trying CXCR1/2 inhibitors as they could have unexpected toxicities. In addition to promoting tumor genesis by increasing angiogenesis and invasion, IL-8 is reported to exert anti-tumor effects through neutrophil recruitment. Hence, CXCR1/2 inhibitors could inadvertently promote tumor growth by blocking the anti-tumor effects of neutrophil infiltration. Novel technologies aimed at delivering drugs specifically to the cancer cells could help minimize these effects. Ultimately, combining CXCR1/2 inhibitors with existing chemotherapy and endocrine therapy agents or IL-8-targeted therapies (or both) may be more effective at eliminating AH MDBs derived HCC.

This breakthrough technology (RNA-Seq) enables us to comprehensively obtain a patient’s overall liver transcriptome data much easier and faster than before. With this information, combined with previous research data, doctors will be able to predict a patient’s response to a specific treatment more accurately, which will finally help realize personalized medicine.
